# Risk factors for death among critically ill patients with acute renal failure

**DOI:** 10.1590/S1516-31802006000500004

**Published:** 2006-09-07

**Authors:** Geraldo Bezerra da Silva, Elizabeth De Francesco Daher, Rosa Maria Salani Mota, Francisco Albano Menezes

**Keywords:** Acute kidney failure, Risk factors, Oliguria, Sepsis, Prognosis, Insuficiência renal aguda, Fatores de risco, Oligúria, Sepse, Prognóstico

## Abstract

**CONTEXT AND OBJECTIVE:**

Acute renal failure is a common medical problem, with a high mortality rate. The aim of this work was to investigate the risk factors for death among critically ill patients with acute renal failure.

**DESIGN AND SETTING:**

Retrospective cohort at the intensive care unit of Hospital Universitário Walter Cantídio, Fortaleza.

**METHODS:**

Survivors and non-survivors were compared. Univariate and multivariate analyses were performed to establish risk factors for death.

**RESULTS:**

Acute renal failure occurred in 128 patients (33.5%), with mean age of 49 ± 20 years (79 males; 62%). Death occurred in 80 (62.5%). The risk factors most frequently associated with death were hypotension, sepsis, nephrotoxic drug use, respiratory insufficiency, liver failure, hypovolemia, septic shock, multiple organ dysfunction, need for vasoactive drugs, need for mechanical ventilation, oliguria, hypoalbuminemia, metabolic acidosis and anemia. There were negative correlations between death and: prothrombin time, hematocrit, hemoglobin, systolic blood pressure, diastolic blood pressure, arterial pH, arterial bicarbonate and urine volume. From multivariate analysis, the independent risk factors for death were: need for mechanical ventilation (OR = 3.15; p = 0.03), hypotension (OR = 3.48; p = 0.02), liver failure (OR = 5.37; p = 0.02), low arterial bicarbonate (OR = 0.85; p = 0.005), oliguria (OR = 3.36; p = 0.009), vasopressor use (OR = 4.83; p = 0.004) and sepsis (OR = 6.14; p = 0.003).

**CONCLUSIONS:**

There are significant risk factors for death among patients with acute renal failure in intensive care units, which need to be identified at an early stage for early treatment.

## RESUMO

**CONTEXTO E OBJETIVO:**

Insuficiência renal aguda é um problema comum e com alta taxa de mortalidade. O objetivo deste estudo é investigar os fatores de risco para óbito em pacientes críticos com insuficiência renal aguda.

**TIPO DE ESTUDO E LOCAL:**

Estudo retrospectivo com pacientes da unidade de terapia intensiva do Hospital Universitário Walter Cantídio, Fortaleza, Brasil.

**MÉTODOS:**

Foram comparados os sobreviventes com os não-sobreviventes, e realizada análise univariada e multivariada, para estabelecer os fatores de risco para óbito.

**RESULTADOS:**

Insuficiência renal aguda ocorreu em 128 pacientes (33,5%), sendo 79 homens (62%), com idade média de 49 ± 20 anos. Óbito ocorreu em 80 (62,5%). Os fatores mais freqüentemente associados ao óbito foram hipotensão, sepse, uso de drogas nefrotóxicas, insuficiência respiratória, insuficiência hepática, hipovolemia, choque séptico, disfunção de múltiplos órgãos, necessidade de drogas vasoativas, necessidade de ventilação mecânica, oligúria, hipoalbuminemia, acidose metabólica e anemia. Foi encontrada correlação negativa entre óbito e tempo de protrombina, hematócrito, hemoglobina, pressão arterial sistólica, pressão arterial diastólica, pH arterial, bicarbonato arterial e volume urinário. A análise multivariada mostrou como fatores de risco independentes para óbito: necessidade de ventilação mecânica (OR = 3,15; p = 0,03), hipotensão (OR = 3,48; p = 0,02), insuficiência hepática (OR = 5,37; p = 0,02), níveis baixos de bicarbonato arterial (OR = 0,85; p = 0,005), oligúria (OR = 3,36; p = 0,009), uso de drogas vasoativas (OR = 4,83; p = 0,004) e sepse (OR = 6,14; p = 0,003).

**CONCLUSÕES:**

Existem importantes fatores de risco para óbito em pacientes em cuidados intensivos, que devem ser prontamente identificados para tratamento precoce.

## INTRODUCTION

Acute renal failure (ARF) is a common medical problem, affecting up to 5% of all hospitalized patients.^1,2^ Among critically ill patients, ARF has a prevalence of 10-30% and a mortality rate ranging from 50 to 90%, depending on the population studied.^1,3-8^

The mortality rate has not significantly changed over recent years, despite advances in the prevention and treatment of ARF.^9^ This may be explained by considering the changes in demographic characteristics, such as the age of patients with ARF, which continues to rise, and the presence of comorbid diseases, which are increasingly common in this population.^1^ The high mortality rate due to ARF among critically ill patients may be a reflection of the severity of the baseline disease, a situation that frequently occurs with multiple organ dysfunction.^10^

Over the past years, several studies have been performed with the aim of identifying prognostic factors and predicting the outcomes for critically ill patients with ARF admitted to intensive care units (ICUs).^7,10-13^ The risk factors for death identified in previous studies were advanced age, male gender, prolonged hospital stay, hepatic or biliary tract diseases, hematological dysfunction, hypotension, shock, coma, need for vasoactive drugs, respiratory distress or need for mechanical ventilation, sepsis, need for dialytic treatment, high levels of creatinine, oliguria and delayed consultation with a nephrologist.^6,8,11,12,14-16^ Better knowledge of factors associated with a bad prognosis in ARF cases is very important for improving prevention and treatment, especially among critically ill patients.

The risk factors for death in ARF cases have not been well established, and the various studies performed on this subject have produced diverse results.^4,6,8-16^ The present study had the aim of investigating the risk factors for death among critically ill patients with ARF admitted to an ICU in northeastern Brazil. Knowledge of these factors will be important for adopting preventive measures and more adequate treatment that could decrease mortality rates.

## PATIENTS AND METHODS

This study was conducted at Hospital Universitário Walter Cantídio, which is part of Universidade Federal do Ceará, in Fortaleza, Brazil. The protocol for this study was approved by the Ethical Committee of the Institution. The records of all the patients admitted to the Intensive Care Unit (ICU) from January 2000 to February 2004 were retrospectively evaluated. All patients who presented ARF during ICU stay were included in the study. ARF was defined as serum creatinine levels greater than 1.5 mg/dl or elevation of more than 50% above the basal value. We excluded patients with chronic renal failure (even those with elevation of baseline creatinine), kidney transplantation, urinary tract obstruction, or hypovolemia that was responsive to fluids within the first 24 hours.

Specific information, such as cause of ARF, comorbidities presented by each patient, need for medications, complications during hospital stay, time for ARF to develop, length of ICU stay, time until starting dialysis and cause of death, were evaluated.

The clinical and laboratory features during ICU stay were studied. Hypotension was defined as mean arterial blood pressure (MAP) < 60 mmHg, and therapy with vasoactive medication was initiated when MAP remained < 60 mmHg. Systolic blood pressure (SBP) and diastolic blood pressure (DBP) at admission were also analyzed. Sepsis was defined according to the American College of Chest Physicians/Society of Critical Care Medicine (ACCP/SCCM) Consensus Conference.^17^ Hepatic dysfunction was defined as serum total bilirubin > 2 mg/dl or any other clinical condition that indicated liver failure; respiratory insufficiency as the need for mechanical ventilation; metabolic acidosis as pH < 7.35 and arterial bicarbonate < 20 mEq/l; and coagulation abnormalities as when the platelet count was < 100 × 10^3^/mm^3^ or prothrombin time (PT) < 65%. Oliguria was arbitrarily defined as urine volume of less than 600 ml/day after appropriate fluid replacement. The other laboratory data evaluated were serum urea and creatinine, total blood count, aspartate amino transaminase (AST), alanine amino transaminase (ALT), serum sodium and potassium, serum albumin and urinalysis.

The patients were divided into two groups: survivors (group I) and non-survivors (group II), in order to investigate whether there were differences in relation to any of the parameters studied. The possible risk factors for death that we hypothesized were the presence of hypotension, sepsis, need for continuous vasopressor drug use (epinephrine, norepinephrine or dopamine), loop diuretics, aminoglycosides, angiotensin-converting enzyme (ACE) inhibitors, need for mechanical ventilation, hypertension, diabetes mellitus, liver failure, cardiovascular diseases, surgery, neoplasms, need for dialytic treatment, time until dialysis started, oliguria, high levels of creatinine (> 3 mg/dl), coagulation abnormalities, metabolic acidosis, anemia (hemoglobin < 10 g/dl), thrombocytopenia and severe hypoalbuminemia (serum albumin < 2 mg/dl).^18^ These had been investigated at ICU admission for all the patients included in the study. The medical records of all the patients were reviewed up to the day of hospital discharge or the occurrence of death (in-hospital mortality).

### Statistical analysis

The results were expressed by means of tables and summary measurements (mean ± standard deviation) in the cases of quantitative variables. The statistical analysis consisted of univariate and multivariate analysis of clinical and laboratory data, which was performed using the SPSS 10.0 software (SPSS Inc., Chicago, USA) and Epi Info 6.04b, 2001 (Centers for Disease Control and Prevention, USA). Comparison of the parameters for the two groups was done by means of Student's t test and Fisher's exact test. Analysis of correlations between death and categorized risk factors was done by means of Fisher's exact test, Pearson's chi-squared test and the verisimilitude ratio test. A logistic regression model was used for quantitative variables. Adjusted odds ratios and 95% confidence intervals were calculated. Multivariate logistic regression was performed to analyze the possible risk factors for death during the first 24 hours after the diagnosis of ARF and during the evolution (beyond this initial 24-hour period). The factors included in the multivariate model were those that showed a significance level < 20% in the univariate analysis (Mann-Whitney test and chi-squared test). Descriptive values below 5% (p value < 0.05) were considered statistically significant.

## RESULTS

Over the period of this study, 381 admissions to the ICU of our hospital were recorded. ARF was detected in 128 cases (33.5%), of which 79 were males (62%). The ages of the ARF cases ranged from 14 to 95 years (mean: 49.5 ± 20.6 years). The mean length of ICU stay was 17 ± 15 days. The time between ICU admission and the development of ARF ranged from one to 36 days (3.21 ± 6.41 days).

The main causes of ARF were hypotension (48.4%), sepsis (40.6%), nephrotoxic drugs (21.9%), rhabdomyolysis (9.1%), hepatorenal syndrome (3.9%), vasculitis (3.1%) and glomerulonephritis (1.6%).

The comorbidities present at ICU admission were respiratory insufficiency (28.9%), cardiovascular diseases (25.8%), hypertension (19.5%), diabetes mellitus (9.4%), surgical complications (9.4%), liver diseases (7.8%) and neoplasms (5.3%). The need for vasoactive drugs, such as norepinephrine and dopamine, was observed in 66 patients (51.6%), loop diuretics in 48 (37.5%), aminoglycosides in 26 (20.3%) and ACE inhibitors in 22 (17.2%). Dialysis was required for 41 patients (32%) and all these patients received intermittent venovenous hemodialysis.

The mortality rate was 62.5% (80 cases). The causes of death were septic shock (51.2%), hypovolemic shock (12.5%), respiratory insufficiency (10%), cardiogenic shock (7.5%), severe metabolic acidosis (7.5%) and liver failure (2.5%).

The patients with sepsis (n = 52) presented a higher mortality rate (82.7%), in comparison with the patients without sepsis (n = 76), whose mortality rate was 48.6% (p < 0.0001). The patients who required dialytic treatment presented a higher mortality rate (41/73.2%), in comparison with the patients who did not require dialysis (87 versus 57.3%), but this difference was not statistically significant (p = 0.11).

The comparison of the clinical and laboratory parameters between survivors (group I) and non-survivors (group II) is shown in Tables 1 and 2. It was found that the ages in group II were significantly greater than in group I. The length of ICU stay, time for ARF to develop following hospital admission and time until dialysis started were also significantly greater in group II. The risk factors most frequently found associated with death among non-survivors were hypotension, sepsis, use of nephrotoxic drugs, respiratory insufficiency, liver failure, hypovolemia, septic shock, multiple organ dysfunction, need for vasoactive drugs, need for mechanical ventilation, oliguria, hypoalbuminemia, metabolic acidosis and anemia. Hypertension was more frequent among survivors. This group presented lower systolic and diastolic blood pressures. ACE inhibitors were used more frequently in group I. The values for urea, AST, ALT, creatine kinase (CK) and total bilirubin were higher in group II, and the prothrombin time and hematocrit and hemoglobin levels were lower in group II.

**Table 1:** Clinical characteristics of critically ill patients with acute renal failure (ARF) admitted to a Brazilian intensive care unit (ICU)

	Survivors (n = 48)	Non-survivors (n = 80)	p
**Age**	46.9 ± 20.7	51.1 ± 20.5	< 0.001
**Sex (male/female)**	32/16	47/33	NS
**Time for ARF to develop (days)**	2.3 ± 5.7	3.7 ± 6.7	0.002
**Time until dialysis started (days)**	2.1 ± 2.0	3.2 ± 3.7	< 0.001
**Length of ICU stay (days)**	16 ± 13	18 ± 17	0.006
**Hypotension**	13 (27%)	49 (61.2%)	< 0.001
**Sepsis**	9 (18.7%)	43 (53.7%)	< 0.001
**Nephrotoxic drugs**	6 (12.5%)	22 (27.5%)	0.04
**Respiratory insufficiency**	7 (14.5%)	30 (37.5%)	0.005
**Hypertension**	14 (29.1%)	11 (13.7%)	0.03
**Diabetes**	5 (10.4%)	7 (8.7%)	NS
**Liver disease**	2 (4.1%)	8 (10%)	NS
**Cardiopathy**	14 (29.1%)	19 (23.7%)	NS
**Surgery**	5 (10.4%)	7 (8.7%)	NS
**Neoplasm**	1 (2.08%)	6 (7.5%)	NS
**Liver failure**	7 (14.5%)	29 (36.2%)	0.008
**Septic shock**	1 (2.08%)	33 (41.2%)	< 0.001
**Mechanical ventilation**	11 (22.9%)	45 (56.2%)	< 0.001
**Venous hydration**	29 (60.4%)	55 (68.7%)	NS
**Vasoactive drugs during ICU stay**	14 (29.1%)	52 (65%)	< 0.001
**Loop diuretics during ICU stay**	20 (41.6%)	28 (35%)	NS
**Aminoglycosides during ICU stay**	6 (12.5%)	20 (25%)	NS
**ACE inhibitors during ICU stay**	15 (31.2%)	7 (8.7%)	0.001
**SBP_min_ (mmHg) at admission**	108 ± 32	79 ± 19	< 0.001
**DBP_min_ (mmHg) at admission**	64 ± 24	45 ± 16	< 0.001
**Multiple organ dysfunction**	0	18 (22.5%)	< 0.001
**Dialysis**	11 (22.9%)	30 (37.5%)	NS
**Oliguria**	14 (29.1%)	51 (63.7%)	< 0.001

NS = not significant; ACE inhibitors = angiotensin-converting enzyme inhibitors; SBP_min_ = minimum systolic blood pressure; DBP_min_ = minimum diastolic blood pressure.

**Table 2:** Laboratory characteristics of critically ill patients with acute renal failure (ARF) at admission to a Brazilian intensive care unit (ICU)

	Survivors (n = 48)	Non-survivors (n = 80)	p
**Urea (mg/dl)**	99 ± 45	103 ± 62	0.003
**AST (IU/l)**	143 ± 272	534 ± 829	< 0.001
**ALT (IU/l)**	97 ± 166	379 ± 916	< 0.001
**Albumin (g/dl)**	2.6 ± 0.6	2.3 ± 0.9	NS
**Creatine kinase (IU/l)**	1890 ± 1942	2199 ± 3098	< 0.001
**Prothrombin time (%)**	65 ± 22	52 ± 23	< 0.001
**Total bilirubin (mg/dl)**	7.4 ± 9.0	9.3 ± 12.3	0.002
**Sodium (mEq/l)**	134 ± 6.1	135 ± 8.7	NS
**Potassium (mEq/l)**	4.0 ± 0.8	4.1 ± 1.0	NS
**Hematocrit (%)**	31 ± 6.5	28 ± 7.1	< 0.001
**Hemoglobin (g/dl)**	10 ± 2.2	9.3 ± 2.5	0.01
**White blood cells (10^3^/mm^3^)**	14,999 ± 6,543	16,421 ± 12,369	< 0.001
**Platelets (10^3^/mm^3^)**	154,452 ± 104,729	160,772 ± 117,479	< 0.001
**Arterial pH**	7.38 ± 0.10	7.29 ± 0.12	NS
**Arterial bicarbonate**	21 ± 10	16 ± 5.8	NS
**Anemia**	28 (58.3%)	60 (75%)	0.04
**Thrombocytopenia**	16 (33.3%)	36 (45%)	NS
**Hypoalbuminemia**	4 (8.3%)	20 (25%)	< 0.001
**Hyperbilirubinemia**	15 (31.2%)	19 (23.7%)	NS
**Hyperkalemia**	1 (2.08%)	6 (7.5%)	NS
**Metabolic acidosis**	11 (22.9%)	51 (63.7%)	< 0.001
**Hematuria**	11 (22.9%)	15 (18.7%)	NS
**Leukocyturia**	14 (29.1%)	15 (18.7%)	NS
**Proteinuria**	11 (22.9%)	11 (13.7%)	NS

NS = not significant; AST = aspartate amino transaminase; ALT = alanine amino transaminase.

The risk factors for death found in the analysis by univariate logistic regression were the presence of hypotension, sepsis, liver failure, septic shock, use of vasoactive drugs, use of mechanical ventilation and oliguria. The presence of hypertension, high levels of prothrombin time, hematocrit, hemoglobin, systolic and diastolic blood pressure, arterial pH and bicarbonate, as well as the use of ACE inhibitors, presented a negative association with death (Table 3). The analysis of the quantitative variables showed a negative correlation between mortality and prothrombin time, hematocrit, hemoglobin, systolic blood pressure, diastolic blood pressure, plasma pH and arterial bicarbonate. This showed that the lower the levels of these variables were, the higher the occurrence of death was (Table 3). No correlations were found between age, time for ARF to develop, time until dialysis started and mortality, according to the logistic regression model.

**Table 3:** Univariate logistic regression analysis for risk factors for death among critically ill patients with acute renal failure admitted to an intensive care unit in Brazil

	OR	95% CI	p
**Arterial pH**	0.0001	0.0001-0.04	< 0.001
**ACE inhibitors**	0.2	0.07-0.5	0.002
**Hypertension**	0.3	0.1-0.9	0.04
**Hemoglobin**	0.81	0.69-0.95	0.01
**Arterial bicarbonate**	0.88	0.81-0.95	0.002
**Hematocrit**	0.93	0.88-0.98	0.01
**Diastolic blood pressure**	0.94	0.90-0.97	< 0.001
**Systolic blood pressure**	0.95	0.93-0.97	< 0.001
**Prothrombin time**	0.97	0.95-0.99	0.02
**Liver failure**	3.7	1.4-9.5	0.004
**Oliguria**	3.9	1.7-8.4	< 0.001
**Hypotension**	4.3	1.9-9.5	< 0.001
**Mechanical ventilation**	4.3	1.9-9.6	< 0.001
**Vasoactive drugs**	4.5	2.0-9.7	< 0.001
**Sepsis**	5.0	2.1-11.9	< 0.001
**Septic shock**	33.7	4.4-256	< 0.001

OR = odds ratio; 95% CI = 95% confidence interval; ACE inhibitors = angiotensin-converting enzyme inhibitors.

The distribution of the patients according to the urine volume levels showed that 65 cases (50.8%) presented diuresis ≤ 600 ml/24h and 90 cases (70.3%) ≤ 1000 ml/24h. Oliguria was present in 14 patients (29%) from group I versus 48 (60%) from group II (p < 0.001). The association between urine volume and mortality showed a negative correlation, according to the linear association test. The occurrence of death increased as urine volume decreased (p = 0.035), as demonstrated in Table 4.

**Table 4:** Correlation between urine volume and mortality among critically ill patients with acute renal failure

Death	Urine volume (ml/24 h)					
	≤ 600			> 600 ≤ 1000	> 1000	
	n	%	n	%	n	%
No	19	29.2%	10	40%	19	50%
Yes	46	**70.8%**	15	**60%**	19	**50%**
Total	65	100%	25	100%	38	100%

Linear association test = 4.46; degrees of freedom = 1; p = 0.035.

The independent risk factors for death shown by multivariate analysis were the need for mechanical ventilation, hypotension, liver failure, low arterial bicarbonate, oliguria, vasopressor drug use and sepsis (Table 5).

**Table 5:** Multivariate logistic regression analysis for risk factors for death among critically ill patients with acute renal failure in a Brazilian intensive care unit

Variables	OR	95% CI	p
Low arterial bicarbonate	0.85	0.76-0.95	0.005
Mechanical ventilation	3.15	1.07-9.25	0.03
Oliguria	3.36	1.35-8.35	0.009
Hypotension	3.48	1.13-10.67	0.02
Vasopressor drug use	4.83	1.65-14.12	0.004
Liver failure	5.37	1.27-22.68	0.02
Sepsis	6.14	1.18-20.10	0.003

OR = odds ratio; 95% CI = 95% confidence interval.

## DISCUSSION

We have conducted one of the first studies on the risk factors for death among critically ill patients with acute renal failure in northeastern Brazil. There are few studies on this subject in our country.^12,19,20^

There are some limitations to this study. Our resources were insufficient to allow full collection of data for calculating disease severity in all patients and were limited to performing all the laboratory tests required. We developed this regression model for predicting the mortality rate among ICU ARF patients using the readily available clinical data.

Acute renal failure (ARF) is an important complication among patients in ICUs, which *per se* increases mortality approximately fivefold.^3,21,22^ The higher mortality rate in this group of patients could be a reflection of the severity of the underlying disease.^10^ In our study, ARF was detected in a considerable number of patients from the ICU (33.5%), and half of the patients were already admitted with ARF. This finding has been observed by others.^23^ We observed a similar prevalence between men and women. Some reports have shown a high prevalence of ARF in males but others have shown that the mortality is similar for men and women.^6,22,23^ The mean age of our patients was around 50 years, i.e. similar to the figures in the literature, which range from 51 to 68 years of age.^23^

The main cause of ARF in our study was hypotension, which represented almost half of our population (48%). This was followed by sepsis (40%) and nephrotoxic drugs (22%). Brivet et al.,^23^ in a prospective study, showed similar results from 282 patients with ARF in an ICU. In their study, they found sepsis in 48%, hemodynamic dysfunction (excluding sepsis) in 32% and toxic injuries in 20%.

Sepsis is one of the main causes of ARF in ICUs and is associated with worse prognosis.^11,18,22,24-27^ Patients with sepsis have generalized arterial vasodilatation, with an associated decrease in renal vascular resistance, which causes renal hypoperfusion and acute renal failure.^26^ The most important measure for preventing ARF in such cases seems to be maintenance of adequate renal perfusion by volume expansion and use of vasoactive drugs.^21,24^ Our findings showed a high mortality rate among patients with ARF associated with sepsis and vasoactive drugs. The patients with sepsis presented a mortality rate of 82%, while the patients without sepsis had a mortality rate of 48%. Our data are similar to those of Neveu et al.,^28^ who also observed a higher mortality rate among ARF patients with sepsis than among patients without this condition (74.5% versus 45.2%).

The majority of our patients presented some comorbidity. The most frequently associated diseases seen in these patients were respiratory insufficiency, cardiovascular diseases and hypertension. Patients from ICUs with ARF frequently present multiple organ failure dysfunctions, which carry a high risk for death.^8^ Its incidence has been estimated in approximately 80% of patients with ARF in ICUs.^27^ In previous studies, it was demonstrated that the greater the degree of organ failure was, the more fatal the outcome was.^12^

In the present study, vasoactive drugs were used in a considerable number of patients (51.6%), as were loop diuretics (37.5%), aminoglycosides (20.3%) and ACE inhibitors (17.2%). The need for vasoactive drugs was significantly associated with death in the multivariate analysis. In recent studies, it has been demonstrated that dopamine is ineffective for preventing and treating ARF.^29^ Low-dose dopamine can also be deleterious because it can induce renal failure in normal-volume and hypovolemic patients.^29^ In the case of noradrenaline, its effects on the kidneys are not well established.^29^ It might improve the glomerular filtration rate and renal blood flow because of increased perfusion pressure.^30^ Some authors have suggested that it can be used in cases of vasodilated circulation with normal or increased cardiac output, which would not affect renal function significantly.^30^

In our study, the need for loop diuretics was not statistically different between survivors and non-survivors, and it was not a risk factor for death according to the univariate and multivariate analyses. Loop diuretics are commonly administered to patients with ARF in order to convert oliguric to non-oliguric ARF. Many studies have not shown any significant benefit from the use of diuretics for ARF cases.^31-40^ In a recent study by Mehta et al.,^41^ it was demonstrated that the use of loop diuretics was associated with a 77% increase in the risk of death and non-recovery of renal function. The severity of ARF might be underestimated when the urine output is sustained, which may lead to a delay in instituting dialytic treatment.^42^

The use of ACE inhibitors in our study was not associated with a high risk of death and was shown by univariate logistic regression analysis to be a protective factor. ACE inhibitors are widely used to treat hypertension, congestive heart failure, and progressive proteinuric renal diseases.^43^ In patients who depend on angiotensin II-mediated efferent arteriolar vasoconstriction for maintaining the glomerular filtration rate, ACE inhibitors can cause pre-renal ARF.^43^

Oliguria was significantly more frequent among non-survivor patients. A significant correlation between urine volume and mortality was found in our series. It was demonstrated that the lower the urine volume was, the higher the risk of death was. Our patients with urine volume greater than 1000 ml/24 h had a better outcome. Therefore, oliguria is an important predictive characteristic of acute renal failure, and worse prognosis in oliguric ARF cases has been demonstrated in many series.^22,23,34-38^

The mortality observed among our patients was high (62.5%) and in accordance with other studies, in which mortality ranged from 50 to 90% among critically ill patients with ARF.^1,8^ Liaño et al.,^22^ in a prospective study, found higher mortality among patients with ARF in the ICU (69.9%), in comparison with patients from clinical (42.8%), surgical (36.3%) and nephrology wards (18.1%). One of the highest mortality rates among critically ill patients with ARF was found in a study performed in India, in which 90% of the patients died.^8^ The main cause of death in our study was septic shock associated with multiple organ dysfunction. It is known that sepsis is considered to be the main cause of death among critically ill non-cardiac patients.^26^ Furthermore, mortality among septic patients may be accounted for by other factors, such as organ dysfunction, including cardiovascular and pulmonary complications.

The patients with fatal outcome presented with more severe illness, were older and showed higher incidence of hypotension, sepsis and respiratory insufficiency. All of these are factors that worsen prognosis. The presence of hypertension was more frequent among survivors, which suggests that this is a protective factor. They also required dialytic treatment and needed vasoactive drugs more frequently. The need for dialytic treatment has been associated with a higher mortality rate (50-70%) than among patients with ARF who do not require renal replacement therapy.^1,10,22^ In our study, the mortality among patients on dialysis (73%) was higher than among non-dialysis patients (57%), but this was not statistically significant. Metnitz et al.^10^ found that mortality among patients who required dialysis was more than four times higher than among non-dialytic patients. This was probably because they presented more severe disease and had higher rates of organ failure. The time until dialysis started was longer among these patients, thus suggesting that early institution of renal replacement therapy is important in decreasing mortality.^10^ It is known that, in cases of acute renal ischemia (pre-renal ARF), the earlier an intervention is instituted, the more favorable the outcome is.^1^

The laboratory analysis showed significantly higher levels of urea, AST, ALT, total bilirubin and CK in patients who did not survive. This group also presented lower levels of hematocrit, hemoglobin and albumin, which reflected the poorer condition of these patients. It is not surprising that non-survivors presented worse laboratory tests. Physicians should be alert to adopting a more careful approach when these abnormalities are present.

In the present study, there were some significant factors associated with death, such as prothrombin time, hematocrit, hemoglobin, systolic blood pressure, diastolic blood pressure, arterial pH and bicarbonate. The lower these values were, the higher the death rate was. This suggests that the occurrence of hematological disorders (thrombotic events, anemia or hemorrhage), hypotension, metabolic acidosis and oliguria is associated with a higher risk of death.

Previous studies have demonstrated that albumin is an important risk factor for death.^44-47^ A recent meta-analysis of 90 studies on critically ill patients showed that each 1 g/dl decrease in serum albumin concentration increased the risk of mortality by 137%.^44^ In another meta-analysis of randomized trials, the administration of albumin to critically ill patients was shown to be important for reducing morbidity in hospitalized patients.^47^ Differing from the literature, our findings showed no correlation between serum albumin levels and death.

There are many studies in which attempts have been made to identify prognostic factors for ARF in critically ill patients. In the present study, multivariate analysis of risk factors for death revealed that the following were important predictors for death: need for mechanical ventilation, hypotension, liver failure, low arterial bicarbonate (metabolic acidosis), hypovolemia, oliguria, need for vasopressor drugs and sepsis. Liaño et al.^22^ showed that oliguria, hypotension, mechanical ventilation and jaundice were associated with higher mortality. Metnitz et al.^10^ observed that some medical interventions were associated with higher incidence of death among ARF patients from an ICU (mechanical ventilation, vasoactive medication, cardiopulmonary resuscitation and treatment of metabolic acidosis/alkalosis). The presence of chronic cardiac failure at admission, late onset of renal replacement therapy and presence of chronic respiratory failure at admission were also associated with higher mortality.^10^ In one of the few Brazilian studies on this subject, the presence of liver and biliary tract diseases, respiratory dysfunction, need for vasoactive drugs and sepsis were found to be prognostic factors.^12^ Mehta et al.,^6^ studying 605 cases of ARF in an ICU, showed that advanced age, male gender, respiratory, liver and hematological failure, oliguria, high levels of creatinine and elevated heart rate were factors associated with high mortality. Sural et al.^8^ showed that the presence of sepsis, associated organ dysfunction, severe renal failure requiring dialysis and prolonged stay in the ICU were associated with increased risk of death. The likelihood of survival was found to be associated with the absence of oliguria, absence of dialysis and absence of ischemic acute tubular necrosis.^16^

These results are in accordance with other studies and will be essential tools in designing and implementing future clinical trials within nephrological critical care in our country.

## CONCLUSION

There are important risk factors for death among patients with ARF in ICUs, which need to be identified at an early stage. Our findings support the view that need for mechanical ventilation, hypotension, liver failure, low arterial bicarbonate, hypovolemia, oliguria, need for vasopressor drugs and sepsis are factors associated with poor prognosis. Identification of these risk factors might enable intensive monitoring and early treatment of symptoms.
